# Phenotyping features in the genesis of pre-scriptural gestures in children to assess handwriting developmental levels

**DOI:** 10.1038/s41598-020-79315-w

**Published:** 2021-01-12

**Authors:** Laurence Vaivre-Douret, Clémence Lopez, Audrey Dutruel, Sébastien Vaivre

**Affiliations:** 1 Faculty of Health, Division of Medicine Paris Descartes, Université de Paris, Paris, France; 2 National Institute of Health and Medical Research (INSERM UMR 1018-CESP), Faculty of Medicine, University of Paris-Saclay, UVSQ, Villejuif, France; 3 University Institute of France (Institut Universitaire de France, IUF), Paris, France; 4 Department of Child Psychiatry, Assistante Publique-Hôpitaux de Paris (AP-HP) Centre, Necker-Enfants Malades University Hospital, Paris, France; 5 Department of Endocrinology, IMAGINE Institute, Necker-Enfants Malades University Hospital, INSERM UMR 1018-CESP, Carré Necker Porte N4, 149, Rue de Sèvres, 75015 Paris, France; 6 Faculty of Society and Humanity, Division Psychology, Université de Paris, Paris, France; 7 National Institute of Applied Sciences, University of Polytechnic, Hauts de France, Valencienne, France

**Keywords:** Health care, Paediatric research

## Abstract

Acquiring writing skills is a long developmental process that is conditioned by both the mastery of the gesture and the spatio-temporal arrangement of characters across the page. While the researches in the literature mainly focused on spatio-temporal and kinematics parameters of tracing letters or words using digitizing tablets, no recent research has previously studied the developmental prerequisites of the organization of handwriting useful for clinical assessment and remediation. Aims of the present study was to investigate and validate the phenotyping of the developmental genesis of pre-scriptural graphomotor gestures among school-aged children in achieving correct handwriting. The subject was examined in depth in an ecological setting similar to school, with the objective of assessing handwriting developmental levels. The pre-scriptural graphomotor task studied was to copy a line of cycloid loops on a paper sheet put on the table. This task was chosen because it reflects the execution of the hand movements from one end of the line to the other and in an anti-clockwise direction, as in handwriting. A new methodological approach was applied incorporating both the maturative evolution of postural-gestural features (video-recorded for analysis in 2D reconstruction) and spatio-temporal/kinematic measures collected with a digital pen connected to an analysis software tool to assess the developmental level and provide an understanding of the phenotypical features of the graphomotor gesture. And we also evidence the concurrent validity of the data in displacements, and the better are the spatio-temporal and kinematic measures. Consequently there are phenotypical features, both postural-gestural and spatio-temporal/kinematic in the developmental genesis of the graphomotor gesture with an easy pre-scriptural task. Typically developing school children from 1st to 5th grade, was collected from elementary schools. Five main patterns of displacement gestures were found for the production of the line of loops with a significant developmental progress from 1st to 5th grade. In addition, significant results in comparisons with spatio-temporal and kinematic age-related normative data were highlighted, associated with the quality of the coordination gesture. Lastly external validity in relation to normative values with the standardized handwriting scale BHK (French adaptation of the Concise Evaluation Scale for Children’s handwriting) showed certain significant correlations with spatio-temporal and kinematic measures and the evolution of the displacement gestures (five patterns) used to draw the loops. The better the motor control of the handwriting gesture, the less variety there is in inter-segmental and joint-scriptural task, enabling handwriting developmental levels to be assessed in screening for handwriting disorders, possibly co-occurring with other learning disabilities, and also useful in clinical decision-making processes for handwriting remediation, or simply to assist handwriting gesture acquisition in elementary school.

## Introduction

Written language is the graphomotor execution of sequential symbols to convey thoughts and communication. Handwriting is a complex movement requiring a neurodevelopmental process that involves several synchronized components between the brain and the muscular segments and joints, and also cognitive abilities (language, mental planning, attention…) and affective factors. However, acquiring writing skills is a long developmental process that is conditioned by both the mastery of the gesture and the spatio-temporal arrangement of characters across the page. However, little is known about the etiology and nature of the specific underlying processes involved in the handwriting gesture (motor and spatio-temporal).

Handwriting disorder is considered to be one of the major public health problems among school-aged children worldwide because it has an impact on academic achievement in school and on professional life for adults. The prevalence rates of handwriting disorder among school-aged children range from 6 to 33%^1,2^ with **s**ignificant interference with academic performance, and often in co-occurrence with learning disabilities. Deficits in handwriting performance commonly occur alongside neurodevelopmental disorders, such as Attention Deficit Hyperactivity Disorder (ADHD)^3^ and Autism Spectrum Disorder (ASD), at about 7–15% in dyslexia^4^ and more often with Developmental Coordination Disorder (DCD or dyspraxia) with incidence rates ranging from 50 to 88%^5–7^.

The literature has resorted to a wide range of terms to qualify handwriting disorders: poor handwriting or handwriting dysfunction, dysgraphia, or poor legibility and/or slow writing speed as assessed by a standardized writing test. The DSM-5^8^ which is the most widely used manual for assigning “Specific Learning Disorder”, where the basic criteria **i**nclude symptoms lasting for at least 6 months, significant interference with academic performance, onset during the school-age years**,** and not accounted for by other disorders. It is described as “Impairment in written expression”, including spelling accuracy, grammar and punctuation accuracy, and clarity or organization of written expression. The term dysgraphia usually refers to a disorder of written language expression in children, which is not described in the DSM-5 but in the previous DSM-IV classification as a learning disability, where writing skills are below the expected standard given a person's age measured on the basis of intelligence and age-appropriate educational level. A recent study^9^ identified different features of handwriting disorders in Developmental Coordination Disorder (DCD) concluding to a proportion of 89% of subjects with poor handwriting and 17% with dysgraphia. These authors’ findings showed that dysgraphia was significantly associated with a high incidence of motor impairments and the presence of minor neurological dysfunction (MND), suggesting a disturbance of the motor pathway, which has never been explored and is confused with motor coordination disorder in all DCD studies^10,11^, who highlighted MND among subjects presenting poor handwriting. Only a few authors^12^ have studied certain isolated patterns, such as the way the pencil is handled or grasped, or the hand and/or wrist position when writing, but never gestural coordination. Indeed, studies on handwriting among school children were mainly interested in the spatio-temporal and kinematics of producing letters or words with digitizing tablets to write with a stylus on the screen or directly with a paper on the screen, seeking to identify the motor processes of handwriting in terms of spatial and temporal performances (e.g. inconsistent letter size and shape, poor alignment, speed…) in comparisons between typically developing children and handwriting disorders^13–17^. The digitizing tablet contains electronics that enable it to detect movement on the screen and translate the movements into digital signals that it sends to the computer. The variability in speed is more difficult to control for children with DCD compared to typically developing children, and there is temporal irregularity in the movements. However, there is a lack of robust tools to assess the quality and legibility of the written product^18^. A systematic literature review found few studies focusing on pre-scriptural development or handwriting processes among school-aged children and they only using spatio-temporal measures comparing typically developing children to a group of handwriting disorder. The comparisons between age groups (6–12 years) were conducted on a digitizing tablet with a stylus, either by assigning the task on a paper of copying ellipses^19^ or using pre-scriptural continuous lines of horizontal^20^. Only one study^21^ with typically developing children using the task of ellipses and horizontal figures-of-eight directly on the screen, and the authors concluded that there was an increase in speed with age, and a better line-drawing quality, suggesting better biomechanical control of the arm and better eye-to-hand coordination. Finally, two study^22,23^ using pre-scriptural figures of loops, one with digitizing tablet comparing cycloid loops to epicycloid loops in particular (clockwise), showed better success among 8–9-year-old children on the epicycloid loops^22^. The second study^23^ used an optical electronic system on the pen and paper concluded that the drawing speed was slower among children with learning disabilities. Thus, the use of pre-scriptural tasks is encouraged to assess children’s developmental levels^20^. The graphic trace and the spatio-temporal or kinematics measures are limited in value, and it is recommended^23^ a more detailed, objective evaluation of the organization of the graphomotor gesture. Unfortunately, there have been no recent studies since the first developmental research on children in 1964^24^. There has only been research on the gestural organization of the calligraphy of cursive handwriting based on the first descriptions in adult^25^ of the spatio-temporal course of handwriting movements. Thus, in order to predict correct acquisition of handwriting, it is first necessary to gain better understanding of the both motor and spatio-temporal prerequisites for handwriting. We set out to identify the phenotypical features in the genesis of pre-scriptural graphomotor gestures among school-aged children enabling a mastery of handwriting, before speculating on life-long neurodevelopmental impairment of handwriting/written expression, such as dysgraphia. We hypothesized that a pre-scriptural task such as copying a line of cycloid loops in an ecological setting makes it possible to observe an evolution in the spatio-temporal and the kinematic gestural measures on the one hand and the evolution of the developmental organization of the graphomotor gesture on the other (postural and inter-segmental coordination of the arm movements) among typical school-aged children from different grades. The choice was to draw with a digital pen on a paper sheet put on the table, a line of cycloid loops from left to right with an anti-clockwise graphic movement that is allied to handwriting, with the same direction of the loops and links between letters. In addition, we hypothesized a correlation between the different parameters (gestural, spatio-temporal and kinematic) and a satisfactory concurrent validity score in relation to a standardized handwriting scale. Thus, we expected to define the phenotyping in the genesis of the features of cycloid loops, which could constitute a pre-scriptural task with good predictive value to assess handwriting developmental levels, and a new tool that is easy to implement in elementary schools.

## Results

### Characteristics of the samples

The first sample (1) included 209 typically developing right-handed school children (Table 1), 86 boys (41%) and 123 girls (59%), aged 6 years 3 months to 11 years 10 months (mean 8.70 ± 1.40).

**Table 1:** Characteristics of the children in the both samples 1 and 2 (N = 331).

	Distribution by grade level of the whole sample (N = 331)					
	1rst grade (n = 58)	2d grade (n (n = 75)	3rd grade (n = 72)	4th grade (n = 64)	5th grade (n = 62)	Total (N = 331)
Age (months) (m ± SD)	80.32 ± 3.95	91.72 ± 4.67	103.08 ± 3.73	113.38 ± 4.40	126.27 ± 6.07	102.99 ± 16.20
Gender (F(n)/M(n))	37/21	47/28	41/31	37/27	34/28	196/135

	Distribution by grade level, sample 1 (N = 209)					
	1rst grade (n = 34)	2d grade (n = 52)	3rd grade (n = 46)	4th grade (n = 39)	5th grade (n = 38)	Total (N = 209)
Age (months) (m ± SD)	81.06 ± 4.02	92.96 ± 4.65	103.76 ± 3.68	113.69 ± 3.99	127.08 ± 6.48	103.47 ± 15.88
Gender (F(n)/M(n))	22/12	31/21	31/15	20/19	19/19	123/86

	Distribution by grade level, sample 2 (N = 122)					
	1rst grade (n = 24)	2d grade (n = 23)	3rd grade (n = 26)	4th grade (n = 25)	5th grade (n = 24)	Total (nN = 122)
Age (months) (m ± SD)	78.46 ± 3.64	88.13 ± 3.47	101.38 ± 3.52	113 ± 6.18	124.08 ± 5.13	101.22 ± 16.96
Gender (F(n)/M(n)	15/9	16/7	10/16	17/8	15/9	73/49
BHK degradation score (m ± SD)	− 0.30 ± 0.65	− 0.43 ± 0.56	− 0.29 ± 0.80	− 0.78 ± 0.95	− 0.59 ± 0.91	− 0.48 ± 0.80
BHK velocity score (m ± SD)	0.64 ± 1.02	0.56 ± 1.07	0.40 ± 0.89	0.54 ± 0.96	0.76 ± 0.62	0.57 ± 0.92

*m* mean, *SD* standard deviation, *F* female, *M* male, *BHK degradation score* the lower the degradation score the better the writing quality.

The second sample (2) included 122 right-handed children (Table 1), 49 boys (40%) and 73 girls (60%), aged 6 years to 11.3 years (mean 8.60 ± 1.50). Thus, 331 children (samples 1 and 2) were enrolled to collect spatio-temporal and kinematic data with a digital pen, and 122 children (sample 2) were video-recorded and underwent the BHK test.

### Postural and gestural features

In order to validate the postural and gestural grid of observations, a preliminary videotaped observation on 25 children was assessed for inter-rater reliability between two assessors, and showed high inter-rater reliability (Cohen’s Kappa = 0.90).

The results of the videotape analysis showed a genesis of the pre-scriptural graphomotor gesture that was in accordance with grade level and developmental age (N = 122) with progressive postural distancing of the head and trunk and a decrease in trunk involvement (Table 2).

**Table 2:**
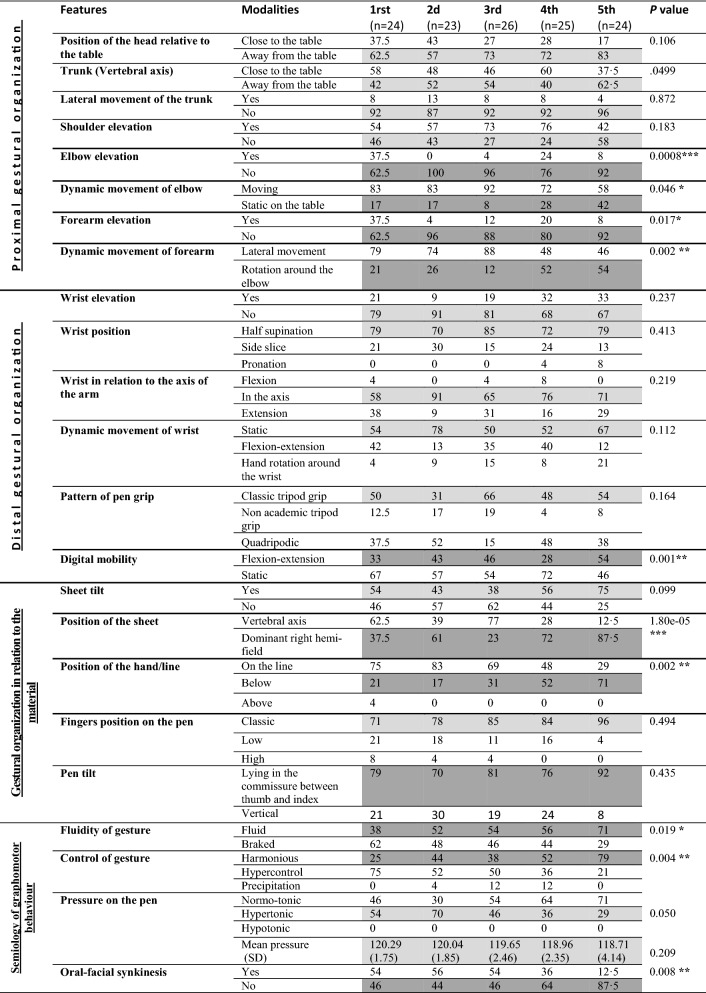
Distribution of the modalities of postural and gestural features according to the grade level (%) in sample 2 (N = 122) for the copy of a line of cycloid loops.

*p < 0.05; **p < 0.01; ***p < 0.001.

Shaded boxes: important frequencies.

*1st* 1st grade, *2nd* 2nd grade, *3rd* 3rd grade, *4th* 4th grade, *5th* 5th grade.

**Figure 1:**
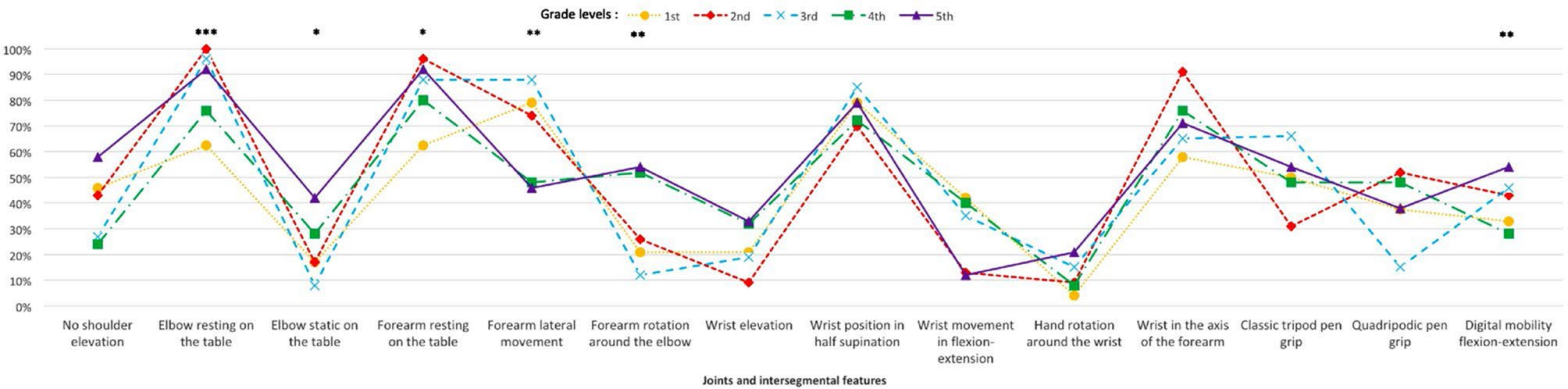
Postural and gestural features of right-handed children drawing a line of cycloid loops. For each grade, frequency of joints and inter-segmental participations of the arm.

In fact, there is variability in the organization of inter-segmental coordination, especially before 3rd grade, and in the elevation of the proximal joints of the writing arm, but this phenomenon significantly stabilizes in 4th and 5th grade (Table 2, Fig. 1).

**Figure 2:**
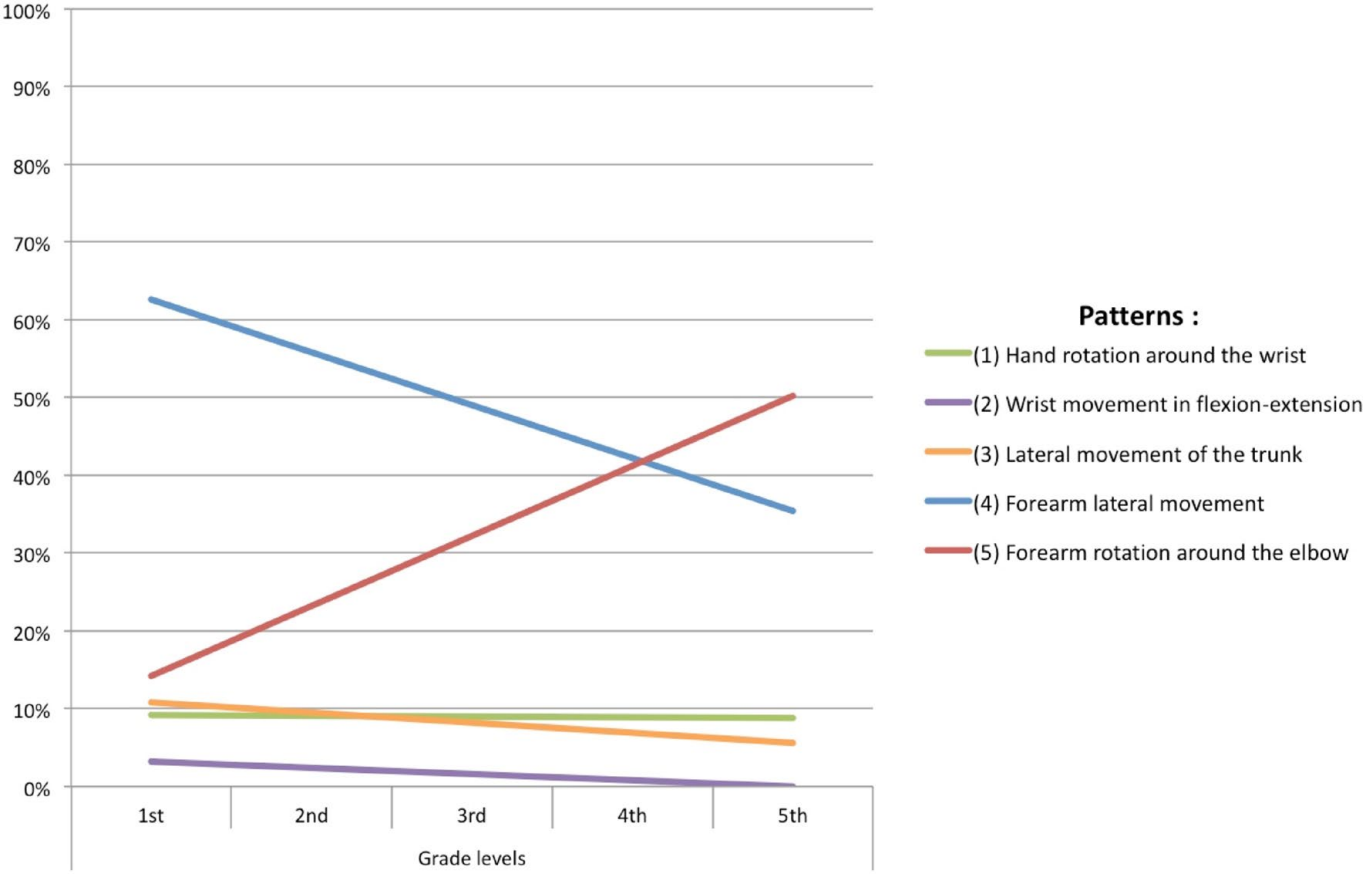
Main patterns (joints and inter-segmental linear regression) of arm movement when drawing a line of cycloid loops. Five significant patterns were described regarding inter-segmental postural and gestural displacements from 1st to 5th grade.

Five main patterns of the displacement gestures required to draw the loops were found according to the grade (Fig. 2): hand rotation around the wrist with a significant synergic inter-segmental coordination of the arm movement in 4th and 5th grade. The use of the pen was mainly tri-digital. There was a statistically significant increase in the numbers of children performing digital flexion–extension movements in 5th grade (*p* = 0.001; Fig. 1).

In addition, there was a significant evolution in the positioning of the sheet of paper, which was located more frequently in the dominant right hemi-field in 5th grade (*p* = 1.80e − 05) associated with right handedness, and significantly often (*p* = 0.002) positioned along the writing line (Table 2).

### Spatio-temporal and kinematic dynamic measures

Concerning the semiology of graphomotor behaviors (Table S1 available online) there was significant correlation with spatio-temporal and kinematic measures: the graphomotor gesture became significantly (*p* = 0.019) more and more fluid from 2nd to 5th grade. The pressure applied to the pen (identifiable by the degree of flexion of the finger joints on the pen) became normo-tonic in 4th (64%) and 5th grade (71%) with a decrease of the mean pressure measured by the pen (Table 2). Oro-facial synkinesis appeared mostly from 1st to 3rd grade and decreased significantly (*p* = 0.008) in 5th grade (12.5%).

Spatio-temporal and kinematic measures from 1st to 5th grade are presented (N = 331) in Table 3.

**Table 3:**
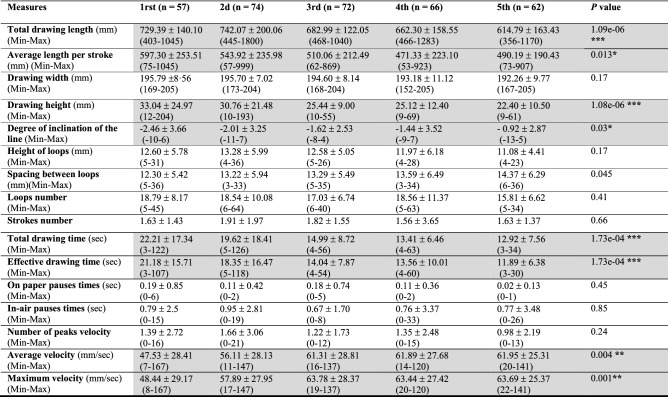
Mean (± standard deviation) of spatial–temporal and kinematic measures by grade level in the whole sample (1 and 2, N = 331) for the copy of a line of cycloid loops.

*1st* 1st grade, *2nd* 2nd grade, *3rd* 3rd grade, *4th* 4th grade, *5th* 5th grade.

*p < 0.05; **p < 0.01; ***p < 0.0001.

Regarding spatial organization, the spacing between the loops tended to increase from 1st to 5th grade. On the other hand, the total length of the line, the height of the figure and the average length per stroke decreased significantly with the grade (*p* = 1.09e − 06, *p* = 1·08e − 06 and *p* = 0.013 respectively), with a decrease in the size of the loops and a decrease in the number of loops in 5th grade. At the same time, the lines became more horizontal, with a significant decrease in the slope (*p* = 0.03). There were fewer acceleration peaks in 5th grade, which indicated better fluidity. On the other hand, the total time spent drawing and the drawing speed decreased regularly with school grades in significant manner (*p* = 1.73e − 04 and *p* = 2.03e − 05, respectively). The averages for the kinematic features "average velocity" and "maximum velocity" increased significantly from 1st to 3rd grade to reach a constant speed from 3rd to 5th grade (*p* = 0.004 and *p* = 0.001 respectively).

### Links between postural and gestural features and spatio-temporal/kinematic measures

Statistical analysis of links between postural and gestural features and spatio-temporal and kinematic measures (presented in Table 4), aimed to determine whether there was a biomechanical influence on the quality of the spatial and temporal data for the loop drawings.

**Table 4:** Results of statistical significant correlations between postural and gestural features and spatial–temporal/kinematics measures in sample 2 (N = 122) for the copy of a line of cycloid loops.

Gestural features	Modalities	Total drawing time (s) m ± SD	Drawing height (mm) m ± SD	Total drawing length (mm) m ± SD	Average velocity (mm/s) m ± SD	Maximum velocity (mm/s) m ± SD
Elbow elevation	No	19.38 ± 10	20.93 ± 8.56	No significant statistics	41.50 ± 15.35	44.20 ± 15.96
	Yes	13.41 ± 7·88	25.06 ± 6.18		57.65 ± 23.47	59.53 ± 22.61
*p* value		0.006**	0.01*		0.007**	0.009**
Forearm elevation	No	19.67 ± 10.1	21.08 ± 8.69	691.04 ± 145.38	41.26 ± 15.35	43.96 ± 16.01
	Yes	12.11 ± 5.67	24 ± 5.88	596.22 ± 93.09	58.17 ± 22.64	60 ± 21.4
*p* value		0.0006***	0.04*	0.008**	0.003**	0.004**
Dynamic movement of wrist	Static	No significant statistics	21.96 ± 8.62	No significant statistics		
	Flexion–extension		23.18 ± 8.18			
	Hand rotation around the wrist		14.92 ± 2.99			
*p* value			0.002**			

	Modalities	Total drawing time (s) m ± SD	Drawing height (mm) m ± SD	Drawing width (mm) m ± SD	Total drawing length (mm) m ± SD	Degree of inclination of the line m ± SD
Displacement of the gesture	Forearm rotation around the elbow	16.24 ± 9.83	20.13 ± 6.61	199.24 ± 5.3	16.95 ± 10.27	-0.82 ± 2.69
	Lateral movement of the forearm and the elbow	18.97 ± 9.09	21.78 ± 8.35	197.12 ± 8.78	20.69 ± 10.32	-1.41 ± 2.96
	Lateral movement of the trunk	16.5 ± 7.93	26.2 ± 8.3	200.8 ± 3.12	16.7 ± 8.01	-3.2 ± 2.3
	Flexion–extension of the wrist	22.5 ± 9.19	45.5 ± 6.36	186.5 ± 12.02	24 ± 7.07	-9 ± 1.41
	Rotation of the hand around the wrist	25.8 ± 14.03	15.9 ± 4.41	201.5 ± 2.88	27.6 ± 14.3	-0.8 ± 1.62
*p* value		0.04*	0.005**	0.03*	0.02*	0.02*

*m* mean, *SD* standard deviation.

*p < 0.05; **p < 0.01; ***p < 0.001.

The features reflecting the proximal segmental gestural organization of the writing arm, "Elevation of the elbow" and "Elevation of the forearm", were significantly associated with variables reflecting the spatial organization of the lines drawn (height (*p* = 0.01 and *p* = 0.04 respectively), total length (for Elevation of the forearm, *p* = 0.008) as well as temporal and kinematic variables of the lines drawn (total drawing time (*p* = 0.006 and *p* = 0.0006, respectively), average speed (*p* = 0.007 and *p* = 0.003, respectively), and maximum speed (*p* = 0.009 and *p* = 0.004, respectively).

In order to know if the task of copying a line of cycloid loops was a good pre-scriptural predictor of, handwriting developmental levels, external validity analyses were conducted using normative values on a standardized handwriting test^26^ first for correlations with spatial–temporal and kinematic measures (Table 5), and then for association with the graphomotor gestural data.

**Table 5:** Statistical significant correlations between BHK scale scores and spatial–temporal/kinematic measures for the copy of a line of cycloid loops (N = 122).

BHK test criteria	Kinetics/kinematics features	Pearson’s *r*	*p* value
Mean total score 11.59, SD 4.80 Range 3.00–26.00	Drawing height (mm)	0.30	0.0009***
	Height of loops (mm)	0.27	0.0051**
Mean number of letters written in 5 mn 203.0, SD 87.97 Range 32–367	Total drawing length (mm)	− 0.18	0.0441*
	Drawing height (mm)	− 0.38	2.046e − 05***
	Degree of inclination of the line (°)	0.25	0.0068**
	Height of loops (mm)	− 0.18	0.0467*
	Total drawing time (sec)	− 0.24	0.0075**
	Effective track time (sec)	− 0.24	0.0064**
	Average velocity (mm/sec)	0.24	0.0088**
	Maximum velocity (mm/sec)	0.25	0.0072**
Criterion 1: writing is too large	Average length per stroke (mm)	0.28	0.0023**
	Drawing height (mm)	0.37	4.639e − 05***
	Degree of inclination of the line (°)	− 0.22	0.0148*
	Height of loops (mm)	0.37	4.29e − 05***
Criterion 2: widening of left-margin	Average length per stroke (mm)	− 0.19	0.039*
Criterion 3: bad letter or word alignment	Drawing height (mm)	0.26	0.0047**
	Height of loops (mm)	0.22	0.0131*
Criterion 4: insufficient word spacing	Total drawing length (mm)	0.22	0.0173*
Criterion 5: acute turns in connecting joins to letters	Total drawing time (sec)	0.24	0.008**
	Effective track time (sec)	0.22	0.016*
Criterion 6: absence or irregularities in joins (break in the trace)	Spacing between loops (mm)	0.21	0.0253*
	Loops number	− 0.21	0.0223*
Criterion 7: collisions of letters	No significant statistics		
Criterion 8: inconsistent letter size (of x-height letters)	Drawing height (mm)	0.25	0.0059**
	Degree of inclination of the line (°)	− 0.18	0.0499*
Criterion 9: incorrect relative height of the various kind of letters	Drawing width (mm)	− 0.21	0.0198*
	Drawing height (mm)	0.24	0.0090**
	Height of loops (mm)	0.20	0.0313*
Criterion 10: letter distorsion	Degree of inclination of the line (°)	0.19	0.04*
Criterion 11: ambiguous letter forms	No significant statistics		
Criterion 12: correction of letter forms	No significant statistics		
Criterion 13: unsteady writing trace	No significant statistics		

*SD* standard deviation.

*p < 0.05; **p < 0.01; ***p < 0.001.

### Concurrent validity with the criteria of the BHK standardized scale and spatial–temporal/kinematic measures

In sample 2 (N = 122), results (Table 5) showed one very significant correlation (*r* = 0.303, *p* < 0.0009): the higher the total raw score for impaired writing on the BHK test, the larger was the size of loops. An even more highly significant result was that the greater the number of symbols copied on the BHK test, the more mature was the spatial organization of the loops. Thus, all the main spatial–temporal and kinematic features were significantly correlated except for criterion 7 (Table 5).

In addition, analyses of the associations between the BHK test and the phenotyping of the graphomotor gesture used for the displacement from one end of the line to the other showed a statistically significant association between immature distal movement along the line (with flexion–extension of the wrist) and the mean size of the loops (*p* < 0.022).

The fewer the written letters subjected to correction on the BHK test (*p* = 0.008), the more mature was the graphomotor phenotyping (gestural displacement by “rotation of forearm around fixed elbow”) with a mean impairment score for this BHK criterion of 0.18/5. Children with a slightly less mature graphomotor gesture phenotyping (displacement with “lateral movement of forearm and elbow”) had a mean score for this BHK criterion of 0.37/5, and children with the most immature graphomotor displacement gesture (“flexion–extension of the wrist”) reached a mean score for this BHK criterion of 1.5/5.

## Discussion

It is well-known that after the scribbling period between 1 and 2 years of age, young children start to draw horizontal lines at the age of 23 months, vertical lines around the age of 29 months, and they reproduce a circle around 32 months^27^. They also acquire the basic spatial notions of top versus bottom and over versus under between the ages of 24 months and 3 and a half years. They start to draw their first lines of loops in infant school and they recognize their right from their left during their first year of primary school. These preliminary acquisitions enable children to easily reproduce a line of loops from left to right drawn in an anti-clockwise direction, as implemented in our study. Indeed, the importance of this task here is that all primary school children are able to carry it out, without requiring prior acquisition of writing and irrespective of interference from associated cognitive disorders, e.g. reading difficulties, thus isolating the particular motor and spatio-temporal prerequisites for this graphism. Our findings show that there is a statistically significant evolution, both qualitative and quantitative (dynamics data), in the execution of graphomotor gestures to reproduce a line of cycloid loops, that follows a significant maturative progression of motor regulation according to grades, and therefore age. Concurrent and content validity were found using the standardized BHK handwriting test^26^ because it is a developmental assessment scale with spatial–temporal criteria, despite that they are static data. The present results thus show that there is a phenotyping (postural-gestural and spatial–temporal/kinematic) in the developmental genesis of graphomotor gestures, and that the pre-scriptural task of reproducing lines of cycloid loops, which is easy to perform whatever the child’s age, and before writing involving spelling rules is introduced, seems to be a good tool that could help to identify the performance levels of the handwriting development. Indeed, this is the first study to our knowledge to have demonstrated that the graphomotor reproduction of a line of cycloid loops, which is above all a process of hand displacement from one end of the line to the other, as with writing (and using an electronic digital pen), shows a developmental maturative evolution in postural and gestural features (5 patterns, Fig. 2). It is associated with a significant evolution in the mean spatial–temporal and kinematic performances of loops drawing, for which we have identified norms according to grades. As shown in Table S1 (available online) and synthesized in Fig. 1, in 1st grade there is greater proximal gestural organization variability, characterized by a significant higher frequency of elevation of the elbow and forearm (37.5% vs < 8% in 5th grade), and a significant lateral movement of the forearm (pattern 4) in the first three grades (79–88% vs 46% in 5th grade) mainly becoming a more significant economic rotation movement around the elbow in 5th grade, with the elbow tending significantly to be static on the table. The distal gestural organization in relation to the wrist position is not significantly different across grade levels, with the majority in half-supine position (≥ 70%) and few in an elevated position. As the flexion–extension wrist movement decreases, it becomes more static with a tendency to remain in the axis of the forearm for more than half of the children in all grades, which helps to control the drawing gesture. The type of grip on the pen is essentially a classic tripod with significantly more marked digital mobility in 5th grade. Thus, there is a progressive developmental evolution whereby the trunk and the head straighten (Table S1, available online) and there is greater stability of the trunk, contributing to better control of graphomotor gestures, according to Miyahara et al*.*^28^, with a significant increase in the stability of the upper limb and joints. This suggests muscular tonic regulation with better dissociation and synergic coordination, involving a reduction in the degree of freedom of the joints for an automatization of more efficient gestures (spatio-temporal measures) from pattern 4 and particularly with pattern 5 “Forearm rotation around the elbow”) around 4th and 5th grade. Indeed, there is a highly significant, strong link (*p* = 2.43e − 16, Cramér’s *V* = 0.72) between a fixed elbow resting on a surface and the use of rotation of the forearm for the drawing movement for the line of loops. There are also links, of medium to strong significance, with the wrist resting on the table and kept in the same axis as the forearm (*p* = 0.0247, Cramér’s *V* = 0.21) and in half-supine position (*p* = 0.0019, Cramér’s *V* = 0.32). This is associated with the classic tri-digital grasp of the pen (*p* = 3.81e − 08, Cramér’s *V* = 0.40). This evolution in gestural organization is the most mature pattern that has been found among adults since Callwaert’s first observations in 1942^25^, and confirms the only descriptions of movement observed on a cursive writing task^24,29^, at a later age (“Forearm rotation around the elbow” between 11 and 14 years old), since writing is a more complex skill, therefore requiring more learning time. Furthermore, in our current study we found spatial–temporal and kinematic components that are highly correlated to this type of gestural pattern (Table 4) showing a developmental evolution taking the form of a significant decrease in drawing time, height of loops, width, total length, and degree of line inclination. Our results underline that motor coordination appears to be a determinant of the drawing quality. Indeed, immature gestures such as "Elevation of the elbow" and "Elevation of the forearm" which are very strongly linked (*p* = 3.483e − 13, Cramér’s *V* = 0.66) are significantly associated spatially, with a larger size of loops and a shorter line of loops, both in temporal and kinematic terms, and with a faster drawing speed. The mean pressure decrease at 4 and 5th grades (Table 2) and the pressure applied on the pen is also correlated to deleterious spatial–temporal and kinematic parameters (Table S1, available online), in agreement with a recent study of Asselborn et al.^30^ using iPad tablet with a pencil to access children’s handwriting difficulties with a numerical approach of the BHK scale. However, this has been studied very little in the literature. Other authors^15,20^, measured it also electronically from the pressure of the pen applied to the digitizing tablet and did not evidence any difference in the mean pressure between a group of children with versus without a writing disorder; it was merely less regular among children with a writing disorder, with or without DCD.

The more immature is the motor control of the handwriting gesture (1st and 2nd grade), the more variety is seen in joint and inter-segmental displacements, which means that there is a learning process in graphomotor skills and an ongoing maturation of the central nervous system (CNS) that can enable the consequences of movement to be anticipated in order to achieve better efficiency. This is in line with the biomechanical constraints in balance control during childhood^31^. Therefore, the more mature is the graphomotor gesture, the more easily is it controlled, with a regulation of the pressure applied and a greater speed of execution. In addition, the maturation of the CNS is characterized by an automatization of movements, a decrease in oral-facial synkinesis (*p* = 0.008) and other significant developmental aspects of graphomotor behavior, such as more fluidity of gesture (*p* = 0.019) in 5th grade (71%) vs 1st grade (38%) and more normo-tonic pressure on the pen (*p* = 0.05) (71% vs 46% respectively (Table S1 available online). Graphomotor patterns are highly correlated (*p* < 0.002) with the progress of spatial–temporal and kinematic features (Table 4). The drawing speed increases sharply from 1st to 3rd grade and stabilizes from 3rd grade. One study^32^ confirmed our results via a task on the letter "e" in different sizes, with a decrease in drawing time between 6 and 8 years followed by relative stability between 8 and 10 years. Another study however^21^ evidenced a constant increase in drawing speed for horizontals, figures-of-eight and ellipses between 8 and 12 years, probably because the task is more difficult to master. One study^22^ compared loop directions and showed better execution of cycloids among 8–9 year-olds, thus highlighting the contribution of learning to trace letters in the same direction.

The current study is the only study that has produced grade-related normative of spatial–temporal and kinematic dynamic values linked to the postural and gestural features in a pre-scriptural graphomotor task among children from different grades (1st–5th). This provides a better understanding of the quality of motor control in achieving good handwriting skills. Indeed, most research in the literature on handwriting has only studied some spatial–temporal/kinematic data, comparing children with or without handwriting disorders^15–17,20^ and they merely concluded to better performances in the group of typical children. In addition, the majority of studies used digitizing tablets to produce letters^13,15,17,32^ or words^14,33^ or for copy tasks; only one used tasks involving ellipses^19,21^ and two involved loops^22,23^.

The concurrent validity of our pre-scriptural graphomotor task is demonstrated by high correlations with results from the standardized BHK writing test^26^ and for the level of maturity of the children’s graphomotor gestures in our sample derived from the spatial–temporal and kinematic data. The more immature is the graphomotor gesture when reproducing a line of loops, the larger the writing in the BHK test and the more often are letters significantly corrected, thus highlighting the specific relevance of observation of the genesis of the graphic movement (gestural patterns) according to age, to identify developmental levels of handwriting. Similarly, the evolution of time-dependent and kinematic measures (total drawing time, pauses during which time the pen is lifted, average speed, maximum speed) is also highly and significantly correlated with the scores on the BHK test for impairment in all writing criteria, and also with the speed of displacement (Table 5). Thus the task of copying of loops linked to grade and age-related normative data appears to be a good predictor for legibility and writing speed. The absence of pauses, correlating with fewer letter corrections in the BHK test, points to a certain fluidity in movement, which helps to gain speed and consequently the automatization of mental preparation in planning the graphomotor gesture, thus showing more mature and proactive motor control^34^ possibly from the 3th grade. These findings concur with the spatial–temporal and kinematic results for children with DCD concerning handwriting, compared to typically developing children^17,30,35,36^ evidencing a larger number of pauses between letters within a word, and corrections. However, another component in our findings that could confirm this graphomotor automatization appears more important, and that is the regularity of space in drawing loops, with a decrease in the number of velocity peaks in 5th grade (Table 3) whereas the number of velocity peaks seems to increase among DCD children^15,17^. Similarly to what is observed in the Pagliarini et al.^37^ study, it appears an inherent rhythmic dimension before handwriting movements turn ballistic and automatized. Hence, in pre-scriptural genesis, there appear to be sensory, visual and motor kinesthesic activities, where hand-to-eye coordination (parietal lobe) reinforces the oculo-motor perceptive activity required for good anticipation for the control of the graphic gesture (basal ganglia) and its timing regulation (cerebellum), suggesting the possible implication of the occipital-parietal-basal ganglia-cerebellar circuit. It could be affected when there is developmental immaturity with motor control of the movement^38^ or in visuo-spatial/constructive DCD, in which children present handwriting impairments^7,39^.

Certain limitations should be considered. Recruitment of typical school-aged children was limited to right-handed children, but they account for at least 90% of the population. It could be interesting to compare our findings with those for a left-handed population. In addition, future research could replicate the current study by a camera-based motion capture system to reconstruct the 3D position of patterns, which would be more accurate. However our recording system with a reference grid provided reliable clinical data with high inter-rater reliability (Cohen kappa 0.90), and reliability is also ensured automatically with the digital pen. In a futur research, it may require more elaborated measures deriving from signal processing. Moreover, it could be examine the predictive validity of our pre-scriptural graphomotor task to identify handwriting difficulties with a group of elementary school children with handwriting disorders.

To conclude, the findings of the present study are an important, original in a developmental perspective, and promising contribution to the construction of normative values for a new tool of graphomotor pre-scriptural test involving elementary school children. The results determined five main patterns in the developmental genesis of the posturo-gestural displacement of the graphomotor gesture, observed from 1st to 5th grade among typically developing children. They proved reliable, and associations were found with normative developmental spatial–temporal and kinematic measures according to grade. Our findings strongly suggest that copying a line of cycloid loops provides an appropriate graphomotor handwriting task for testing school-aged children in ecological settings, consisting in the use of a digital pen similar to the pen used in class and enabling children to write on a standard sheet of paper free to move on the table. Studies in the literature have focused on graphomotor skills using touch-screen tablets to record handwriting, which entails constraints for the children. Indeed, recent studies^40,41^ have demonstrated that drawing directly on the screen of a tablet with a stylus triggered some disturbance in the information process involved in calculating the trajectory of the line drawn, and in muscle and pressure control, and it is required to take also the thickness of the tablet into account. Thus, the description of the genesis of gesture patterns in our pre-scriptural task accompanied by spatial–temporal and kinematic measures can help to identify the level of developmental maturation in the gesture in the perspective of its automatization (motor and spatio-temporal) among children learning to write, so as to detect and explore the nature of the prerequisites of handwriting disorders rather than just being content with handwriting tests and legibility performances. Moreover, it is useful for clinical decision-making processes for handwriting remediation, or simply to assist handwriting gesture acquisition in elementary school.

## Methods

### Participants and design

Data for 331 typically developing right-handed school children aged from 6.1 to 11.0 years old (mean 8.70 ± 1.40) (59% females, 41% males) was collected from elementary schools (grades 1–5) in Paris, France. The subjects were representative of the different socio-professional categories as provided by the French national institute of statistics and economic studies (INSEE).

**Figure 3:**
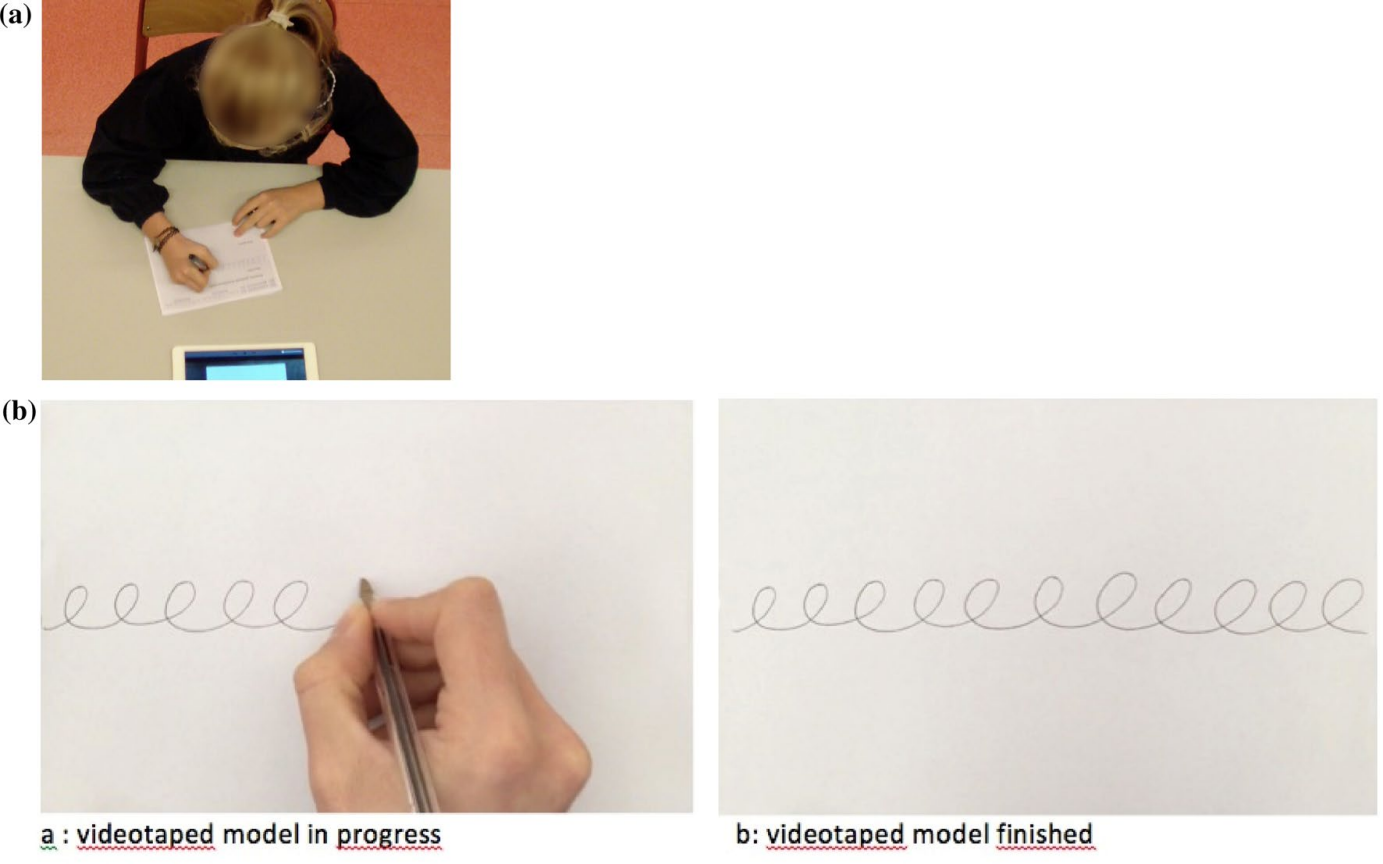
(**a**) Setting with a child (5th grade) when drawing a line of cycloid loops with a digital pen on a paper sheet put on the table. (**b**) Extracted from the videotaped model presented to the child on the iPad tablet for the model of the copy of a line of cycloid loops.

The institutional research ethics committee of Paris Descartes University approved the study procedures (CER·2018-72) performed in accordance with the Declaration of Helsinki. All the parents and the children provided written informed consent. The data collected was anonymised. For the Fig. 3, the consent of the parents has been obtained to publish the image of their child.

The design of study was a prospective, cross-sectional cohort recruited in two phases. Handedness was determined by the hand the child used to write at school.

In the first phase, spatio-temporal and kinematic preliminary data was collected with a digital pen and at the same time postural and gestural observations were made on 209 typically developing school-aged children (sample 1). This was in order to study the feasibility and the validity of the developmental variables. Secondly, a further 122 typically developing school-aged children were recruited (sample 2) to provide spatial–temporal and kinematic measures collected with the digital pen, along with postural and gestural data that was systematically filmed on the basis of the direct gestural observations in sample 1.

### Procedure and measures

An anamnestic questionnaire completed by all parents or legal representatives enabled us to apply the following exclusion criteria to recruit samples of typically developing children: prematurity (birth < 37 weeks of gestation); presence of a neuro-sensory disorder (motor, sensory, visual); brain injury, neuromuscular or genetic disorder; Attention-deficit hyperactivity disorder (ADHD) and Autism Spectrum disorder (ASD) according to the DSM-5 criteria^8^; Specific learning disorder according to DSM-5 (impairment in language and written expression); Developmental Coordination Disorder (DCD); absence of occupational physical therapy; no repeat or skipped grade.

Each child was asked to copy a line of cycloid loops across the width of an A4 size unlined half sheet of white paper.

All children were given the drawing instructions in the same way. They were placed individually in the best possible ecological environment without material constraints, corresponding as closely as possible to a school situation in France. Thus, the posture was standardized: feet flat on the ground or on a stepper, at table height allowing them to rest their forearms on the table without raising their shoulders (Fig. 3a). A model of the dynamic cycloid loop lines was previously recorded and presented to all the children in the same way on videotape (Fig. 3b), with the iPad tablet located on the table in front of the child. The video model was composed of 11 cycloid loops, drawn at a velocity of 0.59 s per cycloid loop. The children were informed that they were free, when copying with the digital pen, to position themselves and to place the paper sheet put directly on the table as they wished. The pen used by the children was a digital pen connected to a handwriting analysis software device. The pen was placed vertically in the middle of the sheet, in line with the body axis, so as not to influence the hand chosen to write. The child was allowed to begin to draw the cycloid loops on the paper sheet after observing the model on the iPad tablet placed in front of him (Fig. 3b).

All children in sample 2 were subjected by the same examiner (psychomotor therapist familiar with BHK to avoid bias) to the French adaptation^26^ of the BHK handwriting assessment test^42^ considered as the gold standard for elementary school-aged children, who are given five minutes to copy a text. It includes an impairment score evaluating the degree of non-legibility, a velocity score, and scores on 13 items measuring different handwriting components. The items relate to the steadiness of the script and to deviations from the conventional forms and positions of letters and words. This enabled us to confirm that only children without handwriting disabilities were included.

In this research, only predominantly right-handed children were recruited to have a homogenous group representative of the population.

Spatial–temporal and kinematic data was recorded for both samples (1 and 2, N = 331) using an Anoto technology electronic digital pen, linked to Elian Research software. We developed the programming algorithms for the specific spatial–temporal and kinematic variables for our analysis. The digital pen is independent from the computer and aggregates and converts written analogue data on paper to digital data. It allows the recording of precise tracks (1/10th of a mm, 1/100th of a second). Its technology is based on a reading by an infrared camera functioning as a barcode and providing the location of the pen on the sheet. The paper contains a unique set of dots printed as a ‘watermark’. Unlike other systems recording tracks on a digitizing tablet, this pen allows the child to be placed in a more ecological situation similar to the French school. Indeed, it provides a normal situation for the children who are free to position their sheet on the table. The pen is light and of medium diameter (about 2 cm of diameter) for easy, comfortable grip. The paper has and invisible grid but a usual appearance so that it does not distract the writer. The children are free to spontaneously organize his graphomotor gesture across the sheet. The data recorded for the organization of the cycloid loop drawings involved spatial (length, size, regularity, slope of the line…), temporal (drawing time, pause time), and kinematics (velocity, peak velocity) measures.

The postural and inter-segmental coordination of arm movements was video-recorded for sample 2 (N = 122) for a 2D reconstruction analysis. Thus, the children were video-recorded using two cameras, one located in front of them and the other located above. These cameras provided a complete and precise view of the child's upper body during the drawing activity. A pre-established direct-observation grid, inspired by the only study with description of the gestural organization in cursive writing^24^, was developed for sample 1. This grid includes criteria concerning the proximal segments and joints (head, axis, shoulder, elbow and forearm) and the distal segments and joints (wrist and fingers) in the coordinated gestural organization of the drawing process, as well as variables reflecting the organization of positioning in relation to their materials (sheet, drawing line, pen). In addition, observational clinical variables on the semiology of the motor characteristics of the (fluidity, control, pressure, synkinesis) were considered.

### Statistical analysis

Analysis were performed using R statistical software (R Core Team, 2019). Frequencies (expressed as percentages) of postural and gestural features were analyzed. The means (SD) for spatial–temporal and kinematic data were calculated. Measures of association between the different features and the grade level were conducted using Chi-squared tests (to analyze postural and gestural qualitative features according to the school grade) or non-parametric Kruskal–Wallis tests (to analyse spatial–temporal and kinematic quantitative features according to the grade). Measures of association between the postural and gestural features and the spatial–temporal and kinematic data were conducted using Mann–Whitney’s paired-sample test (when postural and gestural features had two modalities) or Kruskal–Wallis’s test (when there were more than two modalities for postural and gestural features). Linear regression was performed in Fig. 2 to present graphs of main patterns of the displacement gestures from the grid of video-observations after a detailed descriptions of the postural and gestural features as presented in Table 2 and Fig. 1 . Measures of association between the quality of the gesture for the displacement of the hand from one end of the line of loops to the other, and the BHK test scores were analyzed using Pearson’s correlation test. In addition, Cramér's V was used to measure associations between two nominal variables of gestural organization with a *p* value for the significance of *V* calculated using Pearson's chi-squared test. A fixed Type I error rate of 5% was retained for statistical tests. Two-sided *p* value less than 0.05 were considered significant. There was no missing data.

## Supplementary Information

Supplementary Table S1.
