# Supplementary material for: Phenotyping features in the genesis of pre-scriptural gestures in children to assess handwriting developmental levels
Source: Sci Rep. 2021 Jan 12;11:731. doi: 10.1038/s41598-020-79315-w (PMC7804314; doi:10.1038/s41598-020-79315-w)
Supplement: Supplementary file 1 — Supplementary Table S1. [file 41598_2020_79315_MOESM1_ESM.docx]

**Phenotyping features in the genesis of pre-scriptural gestures in children to assess handwriting developmental levels**

Laurence Vaivre-Douret*, Clémence Lopez, Audrey Dutruel, Sébastien Vaivre

**Prof Laurence Vaivre-Douret**, PhD*

*^1^ Faculty of Health, Division of Medicine Paris Descartes, Université de Paris, France*

*^2^ National Institute of health and Medical Research (INSERM UMR 1018-CESP), Faculty of Medicine, University of Paris-Saclay, UVSQ,Villejuif, France*

*^3^ University Institute of France (Institut Universitaire de France, IUF), Paris, France*

*^4^ Department of Child Psychiatry, Assistante Publique-Hôpitaux de Paris (AP-HP). Centre, Necker-Enfants Malades University Hospital, Paris, France*

*^5^ Department of Endocrinology, IMAGINE Institute, Necker-Enfants Malades University Hospital, Paris, France*

**Clémence Lopez**, PhD

*^1^ Faculty of Society and Humanity, Division Psychology, Université de Paris, France*

*^2^ National Institute of health and Medical Research (INSERM UMR 1018-CESP), Faculty of Medicine, University of Paris-Saclay, UVSQ,Villejuif, France*

**Audrey Dutruel**, MSClin

*^1^ Faculty of Health, Division of Medicine Paris Descartes, Université de Paris, France*

*^2^ National Institute of health and Medical Research (INSERM UMR 1018-CESP), Faculty of Medicine, University of Paris-Saclay, UVSQ,Villejuif, France*

**Sébastien Vaivre**, MSc

*^1^* National Institute of Applied Sciences, University of Polytechnic, Hauts de France, Valenciennes, France

***Correspondence to:**

Prof Laurence Vaivre-Douret, PhD

Hôpital universitaire Necker-Enfants Malades, INSERM UMR 1018-CESP, Carré Necker Porte N4

149, rue de Sèvres, 75015 Paris, France

Tel : +33 (0) 1.44.49.40.14,

[laurence.vaivre-douret@inserm.fr](mailto:laurence.vaivre-douret@inserm.fr)

Table S1. Statistical correlation tests (Kruskal-Wallis ou Wilcoxon) between the semiology of graphomotor behaviour and spatio-temporal /kinematics features (N = 122) for the copy of a line of cycloid loops

| **Graphomotor**  **behaviour** | **Clinical**  **components** | **Stroke number** (SD) | **Average length per stroke** (mm)  (SD) | **Spacing between loops** (mm)  (SD) | **Total drawing time (**sec)  (SD) | **Effective drawing time** (sec)  (SD) | **In-air pauses times** (sec)  (SD) | **Average velocity** (mm/sec)  (SD) | **Velocity**  **Maximum** (mm/sec)  (SD) |
| --- | --- | --- | --- | --- | --- | --- | --- | --- | --- |
| **Quality of the movement** | Fluid  (n = 63) | 1·44  (0.88) | 539.05 (185.36) | 11.43  (3.66) | 13.81 (5.76) | 13.35  (5.10) | 0.32 (1.23) | 53.62 (16.27) | 55.25 (16.15) |
|  | Braked  (n = 55) | 3.44  (3.21) | 380.27 (255.74) | 8.78  (2.39) | 26.64 (11.09) | 24.44 (10.79) | 0.93 (1.80) | 32.64 (11.24) | 36.27 (13.83) |
| ***P* value** | | 2.793e-06  *** | 0.0002  *** | 3.395e-05  *** | 9.233e13  ******* | 2.501e11  *** | 0.0023  ** | 39.363e12  *** | 1.337e09  *** |
| **Gesture control** | Harmonious  (n = 55) |  |  | 11.07  (3.79) | 14.85 (6.83) | 14.07  (6.17) | 0.36 (1.31) | 51.69 (15.65) | 54.13 (15.30) |
|  | Hypercontrol (n = 56) |  |  | 9.29  (2.72) | 25.16 (11.68 | 23.61  (10.92) | 0.73 (1.59) | 33.52 (11.63) | 35.46 (11.79) |
|  | Precipitation (n = 7) |  |  | 10.57  (3.46) | 15.57 (8.46) | 12.71  (4.64) | 1.43 (2.57) | 64.71 (22.67) | 73.29 (17.14) |
| ***P* value** | | N.S. | N.S. | .055 ^NS^ | 2.201e07  *** | 2.736e08  *** | 0.0501^NS^ | 3.174e09  *** | 1.214e10  *** |
| **Pressure on**  **the pen** | Normo-tonic (n = 63) |  |  | 10.89  (3.30) | 16.68 (8.79 | 15.63  (7.73) |  | 47.56 (15.61) | 49.97 (15.78) |
|  | Hypertonic  (n = 55) |  |  | 9.4  (3.34) | 23.35 (11.73) | 21.82 (11.12) |  | 39.58 (18.85) | 42.33 (19.21) |
|  | Hypotonic  (n = 0) |  |  | N/A N/A N/A | | |  | N/A N/A | |
| ***P* value** | | N.S. | N.S. | 0.005 ** | 0.0002 *** | .00002 *** | N.S. | 0.002 ** | 0.004 ** |

** p < 0.01; *** p < 0.001; N.S.: not significant; N/A : not applicable
